# Supplementary material for: Prevention of adolescent stimulant drug use: Do the home life environment and extracurricular activities influence this? Findings from the Irish Planet Youth Survey
Source: PLoS One. 2025 Aug 22;20(8):e0330489. doi: 10.1371/journal.pone.0330489 (PMC12373172; doi:10.1371/journal.pone.0330489)
Supplement: Attachment — Submitted filename: Response to Reviewers 120525.docx [file pone.0330489.s002.docx]

Dr. Karen M. Davison

Academic Editor

PLOS ONE

**Manuscript ID: PONE-D-25-05204**

Dear Dr. Davison,

My co-authors and I would like to thank you and the reviewers for examining our manuscript entitled "Prevention of adolescent stimulant drug use: What are the roles of home life environment and extracurricular activities? Findings from the Irish Planet Youth Survey" and for giving us the opportunity to revise and resubmit our manuscript to your journal.

We have addressed all of the comments and incorporated changes throughout. We believe that our paper has been improved as a result of these modifications. It is with this in mind that we hope the manuscript can now be reconsidered for review and subsequent publication in PLOS ONE.

Yours sincerely,

Fionn Daly

Seán Millar

Emmet Major

Peter Barrett

Corresponding author email: [peter.barrett@ucc.ie](mailto:peter.barrett@ucc.ie)

**Point by point response**

**Reviewer #1**

This manuscript effectively highlights important findings on adolescent stimulant use in Ireland and its associations with parental rule-setting, monitoring, intergenerational closure, and extracurricular activities participation. With some clarifications, streamlined explanations, and stronger policy recommendations, it could have a greater impact on public health discourse.

**Key strengths**

**Robust dataset**

The study draws from a large sample size (n=~5000) with a high response rate, enhancing generalizability.

**Novel insights**

It provides new evidence on intergenerational closure as a potential protective factor against adolescent stimulant use.

**Policy relevance**

Findings can inform family-based and extracurricular interventions in Ireland, especially considering the high cocaine use prevalence among Irish adolescents.
We thank the reviewer for their careful consideration of our manuscript.

**Strengthen justification for the study**

The study compares Irish data with Icelandic trends. It may be useful to briefly mention why Iceland’s model is particularly relevant.

We thank the reviewer for their comment. We have now expanded the Introduction section to explain why the Icelandic Prevention Model (IPM) is relevant to this research. Specifically, we highlight how the IPM underpins the Planet Youth initiative, which was the basis for the data collection in Ireland. Moreover, the IPM offers a framework for examining community and familial factors which may be associated with adolescent substance use.

Highlight why stimulant use trends in Ireland may differ from those in other European nations.

We appreciate the reviewer’s suggestion to clarify why stimulant use trends in Ireland may differ from those observed in other European nations. Although likely multifaceted and complex, we have briefly discussed this point in the Introduction section. These include a cultural shift in the perceived harms of stimulant drugs (particularly among young people), as well as the use of cocaine with alcohol consumption. Internationally, Ireland has a disproportionately high level of alcohol consumption, and this may partially explain higher levels of cocaine use among Irish adolescents compared to other European nations (Ireland ranks second in terms of cocaine use, according to ESPAD survey data). We hope these additions provide greater context for the trends discussed in our study.
**Results
Data interpretation**

Some findings (e.g., sports participation being protective) are expected, but the lack of significant association for volunteering and arts deserves further explanation.
We have now elaborated on this point in the Discussion section. Specifically, we note that the proportion of adolescents engaged in activities other than sports (e.g. arts and volunteering) was much lower, which may have limited statistical power to detect true associations. Thus, it is possible that the lack of observed associations may not necessarily reflect a true absence of effect, but rather a potential limitation related to sample size.

**Table presentation**

Consider highlighting statistically significant findings in bold for clarity. Ensure consistency in decimal places across all tables.

We have now highlighted statistically significant findings in bold and have fixed errors with decimal places in our tables. We thank the reviewer for pointing this out.

**Methods
Define variable more clearly**

The operationalization of intergenerational closure is unclear. A more precise definition or detailed description and explanation of its measurement would be beneficial.
We appreciate the reviewer’s comment regarding the need to more clearly define the concept of intergenerational closure. We have added a concise definition to the Abstract to ensure early understanding for readers. Furthermore, in the Methods section, we have elaborated further, clarifying its definition as the degree of connection, trust and communication between adolescents, their parents, and the parents of their peers. We have also expanded the explanation of how this composite variable was analysed in our sample, including details on the specific survey items used to create the variable, and the scale development process thereafter. We hope this now provides a clearer account of how intergenerational closure was both conceptualised and measured in our study.

Parental rule-setting and monitoring could be elaborated with specific examples.
Specific examples have now been added to the Methods section under “Independent variables”.

**Extracurricular activities**

Since participation is self-reported, consider discussing the potential for social desirability bias.
This has now been discussed in the “Strengths and limitations” section of the Discussion.

The study only examined frequency of participation. Would depth (e.g., competitive vs. Recreational/casual sports) influence the results?

We thank the reviewer for this valuable suggestion regarding the depth or intensity of sports participation. While the survey did not include specific measures of competitiveness or intensity, we have tried to address this limitation by incorporating an additional variable of interest that captures regular sports participation **outside** of a club or team (i.e. informal or casual play). This allows us to partially assess the potential impact of less structured, non-competitive sports engagement, and we observed similar findings to those seen with competitive sports.

**Limitations**
**Self-report bias**

While acknowledged, this could be expanded with suggestions for mitigating this bias in future studies.

We thank the reviewer for highlighting the issue of self-report bias and the importance of addressing it in future research. In our Limitations section, we have included potential strategies for mitigating such bias in subsequent studies. Specifically, we note that future iterations of the survey in Ireland will be conducted digitally, allowing for real-time flagging of inconsistent responses to enhance data validity. Additionally, for some variables such as parental monitoring and rule-setting, we suggest that future studies may consider triangulating adolescent self-reports with data from parents or guardians to provide a more comprehensive perspective. We hope these changes are acceptable to the reviewer.

**Causal limitations**

Emphasize that cross-sectional studies do not establish causation and suggest longitudinal approaches for future research.

This suggestion has now been added to our Limitations section.

**Potential sample bias**

It is stated that 100% of schools in the region participated, but does this sample reflect national trends?

We acknowledge that our sample may not be nationally representative of adolescents in Ireland. However, in the first paragraph of the Discussion section we note that the lifetime prevalence of cocaine use among our study sample was 3.4%, while the lifetime prevalence of ecstasy use was 2.8%. These figures are almost the same as those observed in the European School Survey Project on Alcohol and Other Drugs (ESPAD) report from 2019 (3.3% for lifetime cocaine use and 2.8% for lifetime ecstasy use), which was a nationally representative study. We also note that, despite the predominantly rural population in the West of Ireland, people living in this region exhibit many similar sociodemographic characteristics in comparison to the broader Irish population, and thus, these findings are likely to be generalisable to adolescents elsewhere in Ireland.

**Conclusion**
The conclusion restates findings but could provide a stronger call to action regarding policy implementation.

We appreciate the reviewer’s suggestion to strengthen the manuscript by offering a clearer call to action regarding policy implementation in the Discussion, and we briefly reiterate this in the Conclusion. Specifically, we have highlighted an evidence-based policy initiative from Iceland, known as the “Recreation Card”, which has successfully supported adolescent engagement in structured extracurricular activities and forms a core component of the IPM. We also draw attention to a similar proposal that has been put forward in Ireland (i.e. a “Leisure/Recreation Card”), but which has not yet been implemented. We hope this addition provides a more impactful and policy-oriented conclusion to the manuscript.

Consider adding specific recommendations for intervention strategies rather than just emphasizing the need for further research.

Thank you for this suggestion. As discussed above, we have included further information in the manuscript on our specific recommendation that Irish policymakers consider the introduction and implementation of a subsidised “Recreation Card” for adolescents to enhance their access to structured extracurricular activities. Moreover, a specific, school-based intervention that is now being implemented nationally in Ireland has been incorporated into our Discussion (and reflected upon in the Conclusion). This intervention is the mandatory inclusion of “Social, Personal and Health Education” (SPHE) at both primary and secondary school levels. This curriculum is designed to support development in areas such as emotional well-being, responsible decision-making (including decisions surrounding drug use) and digital awareness. Given the increasing role of online platforms in facilitating access to illicit substances, mandatory SPHE may play a key role in equipping adolescents with the skills needed to navigate these emerging public health challenges. We believe our manuscript has been strengthened as a result of these changes.

**Reviewer #2**

Authors report on the study examining the cross-sectional relationship between indicators of home life and community activities among youth and use of cocaine and/or ecstasy among high school students in Western Ireland. In a sample of approximately 4900 high school students, the current study identifies that approximately 3.4% of Irish high school youth have used cocaine and 2.8% have used ecstasy. Associated risk factors include a lack of parental rule setting, a lack of parental monitoring, and reduced intergenerational closure, defined as how well parents know the peers their children spend time with and the peers’ parents. In addition, students who did not participate regularly in sports were at greater risk of previous cocaine and/or ecstasy use. The study uses a survey adapted from research that informed a successful intervention program developed in Iceland which led to diminished use of illicit substances among you in the early 2000s.

We thank the reviewer for their careful consideration of our manuscript.

**Concerns**
The underlying theoretical model or rationale for choice of indicators regarding home life, community activities, etc. should be more clearly specified in the introduction.

We thank the reviewer for their comment, and we have now clarified the theoretical rationale which guided the selection of indicators related to the home life environment and engagement in extracurricular activities. As now mentioned in the manuscript, the IPM is grounded in the Theory of Social Deviance, suggesting that adolescent substance misuse is a product of the broader social environment rather than purely individual choice. Therefore, the IPM targets key domains in adolescents’ environments, including within the home and community. This has informed our focus on parental rule-setting, monitoring, intergenerational closure and extracurricular activities, all of which are emphasised within the IPM as modifiable risk or protective factors. We hope this provides clearer justification for the inclusion of these variables.

The rationale for presenting the data as two separate but parallel studies is not clear. Presumably the same sample were used for all the data. Can the study be presented as a single data set with two outcomes in this way authors could also present information regarding the youth who used both kinds of illicit stimulants.

We appreciate the reviewer’s comment regarding the rationale for analysing cocaine and ecstasy use as separate, parallel outcomes rather than a single dataset with multiple categories. This was considered *a priori*. However, we consulted with experts in this field, and although both outcomes were derived from the same survey and study population, we opted to analyse them separately for the following key reasons: Firstly, given that stimulant use was relatively uncommon in the sample (3.4% for cocaine and 2.8% for ecstasy), combining these into a multi-category outcome (e.g. “cocaine only”, “ecstasy only”, “both”, “neither”) would have led to small cell counts in several subgroups, potentially affecting the robustness of our analysis due to a loss of statistical power. Secondly, although both are stimulant drugs, cocaine and ecstasy/MDMA tend to have different patterns of use (i.e. they may be used in different social contexts). For example, cocaine is often used in conjunction with alcohol and may be increasingly integrated into more routine social interactions (e.g. bars, parties). Ecstasy/MDMA, by contrast, has traditionally been linked to more specialised settings (e.g. raves, electronic dance music (EDM) events). However, we acknowledge that these distinctions may be evolving. Ecstasy use among young people may not be as confined to such specific settings, and future research could explore whether the social contexts of use have shifted. We hope this rationale is acceptable to the reviewer.

The details of the sample (age/grade, proportion female) are not presented clearly in the abstract or in the initial description of the sample.

These sociodemographic details have now been added to the Abstract and to the Descriptive Statistics section of our Results.

Did the demographic question clearly inquire about gender or sex at birth? Was non-binary an option?

We thank the reviewer for raising this important point regarding the measurement of gender in our study. The demographic question in the survey asked participants about their current gender identity, rather than sex assigned at birth. Response options included "Male", "Female", "Non-binary" and "Prefer not to say". In our analysis, we grouped participants who selected “Non-binary” and “Prefer not to say” into a single category. This was done in an effort to best preserve participant anonymity, as the small number of respondents in each of these groups could otherwise pose a risk of indirect identification.

The stated study objective was to estimate prevalence of cocaine and ecstasy use among Irish adolescents using data from the Irish planet youth 2020 survey. However, it does not appear that estimating a population prevalence was what the study was designed to accomplish. Rather authors reported data from a community sample of youth who attended schools and who answered the survey questions. The study objective needs to be more focused on the goals of examining associations between specific home life factors, organized part extracurricular activities, and the use of illicit stimulants.

We appreciate the reviewer’s comment and agree that this study is best characterised as an analysis of associations within a community sample, rather than a population-level prevalence study. Thus, we have revised the wording at the end of the Introduction to more clearly reflect the study’s primary objective, i.e. to examine associations between specific home life factors, participation in extracurricular activities, and adolescent stimulant drug use. We hope that this revised version more accurately conveys the intent and scope of our analysis and is acceptable to the reviewer.

How closely linked is the study with the research program in Iceland?

We appreciate the opportunity to clarify the relationship between this study and the research programme in Iceland. While the Planet Youth initiative in Ireland is based on the IPM, the study presented here was conducted independently and is not part of a formal research collaboration with the Icelandic group. There is no direct oversight from researchers in Iceland. However, the *Icelandic Centre for Social Research and Analysis* designed the survey and facilitated the data cleaning process through contractual agreement. Setting research questions, data analysis and manuscript preparation were all carried out independently by the authors of this study.

How many Irish youth do not attend school in these counties? Who is missing from the sampling frame?

We thank the reviewer for their comment. While the survey achieved a high response rate (80%) across all post-primary schools and Youthreach centres in the three participating counties, it is likely that a proportion of adolescents were not captured due to absenteeism on the survey day, which may have included reasons such as illness, sports events, or other off-site commitments. However, some adolescents in the region may not be enrolled in formal education and thus fall outside the sampling frame. Unfortunately, we do not have access to official statistics on the exact proportion of youth who do not attend school in this region, although anecdotally we have been advised by school principals that this number is low. We have now acknowledged this in the Limitations section of our manuscript, and hope it is acceptable to the reviewer.

What response options were available for the questions measuring lifetime cocaine use or ecstasy?

We thank the reviewer for their comment. In the survey, participants were asked how many times they had used cocaine or ecstasy/MDMA in their lifetime, and response options included: “Never”, “1–2 times”, “3–5 times”, “6–9 times”, and “10–19”, "20–39", "40 times or more". However, the number of respondents in the higher frequency categories was very small, which limited the feasibility of maintaining a multi-category analysis. Additionally, the survey did not include questions on recency of use (e.g. past 30 days or past year). Therefore, to ensure adequate statistical power, we dichotomised the responses into a binary variable, i.e. lifetime use (yes/no).

The term “intergenerational closure” is new to this reader. The definition could be included in the introduction.

The definition has now been included in the Introduction section.

I suggest referencing/including previous Irish studies of substance misuse using the same sample in the introduction. Were there similar findings with home life variables?

We thank the reviewer for this helpful suggestion and have now referenced previous Irish studies using Planet Youth data in the Introduction section of our manuscript. These studies examined associations between extracurricular activity involvement and the home life environment, and their associations with adolescent cannabis use, as well as binge drinking. We believe this addition further strengthens the rationale for our focus on stimulant use herein.

Please clarify the information that the planet youth research offers in distinction to that provided by the EMCDDA.

We thank the reviewer for raising this point and for the opportunity to clarify the distinction between data provided through the Planet Youth initiative and that produced by the European Union Drugs Agency (EUDA), formerly the European Monitoring Centre for Drugs and Drug Addiction (EMCDDA). The primary aim of the Planet Youth initiative is to support the implementation of the IPM through the collection of community-level data, which focuses on modifiable social and environmental factors which may be associated with adolescent substance use. The information gathered is intended to guide local primary prevention strategies by informing stakeholders, such as schools, community groups, and policymakers, about the lived realities of adolescents within specific regions. In contrast, the EUDA focuses on national and cross-country surveillance by providing internationally comparable statistics, including substance use prevalence, drug-related treatment admissions and overdose deaths. While data compiled by the EUDA are critical for assessing overall trends across Europe, they do not provide the granular, community-specific data needed to inform local, primary prevention-oriented interventions that Planet Youth is designed to support.
