# Supplementary material for: Prevention of adolescent stimulant drug use: Do the home life environment and extracurricular activities influence this? Findings from the Irish Planet Youth Survey
Source: PLoS One. 2025 Aug 22;20(8):e0330489. doi: 10.1371/journal.pone.0330489 (PMC12373172; doi:10.1371/journal.pone.0330489)
Supplement: Attachment — Submitted filename: Response to Reviewers.docx [file pone.0330489.s003.docx]

Dr. Karen M. Davison

Academic Editor

PLOS ONE

**Manuscript ID: PONE-D-25-05204R1**

Dear Dr. Davison,

My co-authors and I would like to thank you and the reviewers again for examining our manuscript entitled "Prevention of adolescent stimulant drug use: What are the roles of home life environment and extracurricular activities? Findings from the Irish Planet Youth Survey" and for giving us the opportunity to revise and resubmit our manuscript to your journal.

We have addressed all of the comments and incorporated changes throughout. We believe that our paper has been improved as a result of these modifications. It is with this in mind that we hope the manuscript can now be reconsidered for review and subsequent publication in PLOS ONE.

Yours sincerely,

Fionn Daly

Seán Millar

Emmet Major

Peter Barrett

Corresponding author email: [peter.barrett@ucc.ie](mailto:peter.barrett@ucc.ie)

**Point by point response**

**Journal Requirements**

Please review your reference list to ensure that it is complete and correct. If you have cited papers that have been retracted, please include the rationale for doing so in the manuscript text or remove these references and replace them with relevant current references. Any changes to the reference list should be mentioned in the rebuttal letter that accompanies your revised manuscript. If you need to cite a retracted article, indicate the article’s retracted status in the References list and also include a citation and full reference for the retraction notice.

Thank you for bringing this to our attention. The link to the previously cited reference, “Western Regional Drug and Alcohol Task Force and the Department of Public Health, HSE West. Planet Youth Survey: Galway, Mayo, and Roscommon, 2020. Irish Social Science Data Archive. SN: 0071-02. 2022. Available at: <https://www.ucd.ie/issda/data/planetyouth/>” is no longer active. In response to the Journal’s guidance, we have replaced this reference with a more current and relevant source. We appreciate this constructive feedback.

**Additional Editor Comments**

Please align definitions of intergenerational closure provided in the abstract and footnoted in your results tables

We thank the editor for their helpful suggestion, and we now have revised the manuscript to ensure consistency in the definition of intergenerational closure across the abstract and the footnotes of the results tables.

Acknowledge limitations of your study in the abstract

Thank you for this helpful suggestion. The abstract now includes key limitations of the study, such as its cross-sectional design, reliance on self-reported anonymised data, as well as the exclusion of adolescents not enrolled in formal education.

Please confirm if mental health diagnosis e.g. depression, anxiety was available in the survey. If it was, please include in analysis. If not, please highlight that this was an important covariate you were unable to control for in your limitations

We appreciate this important point. The survey did not include clinical diagnoses of mental health conditions such as depression or anxiety. However, it did include a self-rated measure of overall mental health, and we have now incorporated this variable of interest throughout the analyses and an interpretation of results in our second round of revisions. Importantly, adolescent mental health is likely influenced by the home life environment, making it a potential mediator in the pathway between home life factors and stimulant drug use. Consequently, adjusting for mental health is not appropriate, as the aim of our study was to estimate the total effect of home life factors on stimulant drug use. In order to avoid “over-adjustment bias”, which may attenuate or obscure true associations, by blocking part of the causal pathway, we have instead examined the mental health variable as an independent predictor of stimulant drug use in our analyses. We hope this clarifies our analytical approach and is acceptable to the editor. It should be noted that examining the causal pathway between the home life environment and adolescent drug use, and how this may be mediated by adolescent mental health, would be an interesting research topic. However, that was not the aim of this study.

**Reviewer #2**

Thank you for your responses, and for the clear explanation as to why the study was completed as two separate analyses. I suggest including a brief version of this rationale in the statistical analysis section of manuscript.

We thank the reviewer for their suggestion. We have now added a brief paragraph of the rationale for conducting the study as two separate analyses. This has been added to the beginning of the Statistical analysis section.

In the results section, description of the rates of cocaine and ecstasy use (as well as other variables) are offered as percentages. Please include confidence intervals for these estimates of percentage.

All included estimates are now followed by 95% confidence intervals in the Results section.

Suggest that Tables 1 and 2 can be simplified by offering only a single value for the yes/no binary variables, e.g. can present only yes, as the no is understood.

We agree that Tables 1 and 2 can be simplified by offering a single value for dichotomous variables, and we have revised the manuscript to reflect this. We thank the reviewer for this suggestion.

The statement regarding combined cocaine and ecstasy should appear in the results rather than in discussion for the first time.

Thank you for pointing this out. The statement regarding the combined reporting of cocaine and ecstasy use now appears first in the Results section, rather than the Discussion.

In the first paragraph of the discussion, do you have a comment about other countries that have looked at this question regarding home life environment and extracurricular activities and stimulant drug use (in addition to Iceland).

Thank you for this suggestion. We have clarified in the first paragraph of the Discussion that, to our knowledge, our study is the first study to specifically examine both home life environment and extracurricular activity variables in relation to adolescent stimulant drug use, both in Ireland and internationally. This highlights the novelty of our contribution, given the paucity of existing research on this specific combination of independent and dependent variables.
